# Challenges of Serum Creatinine Level in GFR Assessment and Drug Dosing Decisions in Kidney Injury

**DOI:** 10.34172/apb.42345

**Published:** 2024-12-08

**Authors:** Xinyi Wang, Jing Mu, Kexin Ma, Yanrong Ma

**Affiliations:** ^1^The First School of Clinical Medicine, Lanzhou University, Lanzhou, 730000, China; ^2^Department of Pharmacy, the First Hospital of Lanzhou University, Lanzhou 730000, China

**Keywords:** Creatinine, GFR, Drug dosing adjustment, Biomarker, Kidney injury

## Abstract

Serum creatinine (SCr) is widely regarded as a standard biomarker for assessing glomerular filtration rate (GFR) and is commonly used to guide dose adjustments for renally eliminated drugs. However, the application of SCr as a marker for evaluating GFR and drug dosing in kidney injury has significant limitations that are often overlooked in clinical practice. This oversight can result in subtherapeutic drug concentrations or adverse drug reactions due to inappropriate dosing adjustments based on SCr levels alone. This review aimed to highlight the factors affecting serum creatinine (SCr) and the challenges associated with using SCr as a biomarker for assessing GFR and adjusting drug doses with regard to its limitations and variability. The findings of this review underscore the complexity of SCr regulation, which is affected by its synthesis, metabolism, and excretion processes (glomerular filtration, tubular secretion, tubular reabsorption and extra-renal elimination), and disease states (such as trauma-induced hyperfiltration and HIV) and the use of medications (drug-creatinine interactions) lead to altered renal excretion of creatinine, either increasing or decreasing its levels. Additionally, the renal excretion pathways for drugs and creatinine are not entirely the same, making it difficult to use creatinine to evaluate drug renal excretion. In conclusion, SCr is an imperfect index of GFR and adjusting drug dosing, and the development of multi-biomarker panels, incorporating biomarkers from different excretory pathways-particularly those involving tubular transport-holds promise for improving the evaluation of renal excretory function and ensuring safer and more effective drug dosing.

## Introduction

 The kidney plays a crucial role in facilitating the excretion of numerous drugs and their metabolites from the body. The dysregulation or decompensation of kidney function may directly affect the pharmacokinetics, pharmacodynamics or toxicity of drugs. Glomerular filtration rate (GFR) represents the overall filtration rate of the functioning nephrons, and is therefore considered the optimal method for measuring overall kidney function and making disease diagnosis decisions^1^

 Creatinine-based estimation of GFR has served as the primary approach for assessing kidney function and adjusting drug dosages.^2^ In 1847, Liebig discovered heating creatine with mineral acids formed a new substance, which he named creatinine. In 1886, Jaffe observed a creatinine reaction with picric acid in an alkaline medium, and this method, known as the Jaffe reaction, was used for measuring creatinine in clinical laboratories until the early 21st century.^3^ Due to the fact that creatinine precursors are synthesized by the liver, creatinine was considered a product of nitrogen metabolism at the time of Jaffe’s discovery. In 1926, Rehberg demonstrated that creatinine was eliminated into the urine via glomerular filtration and was neither secreted nor reabsorbed, thus proposing creatinine as a biomarker of GFR.^4^

 Although measuring the renal clearance rate of exogenous biomarkers such as inulin, 99mTc-diethylenetriamine pentaacetic acid, ^125^I-othalamate and ^51^Cr-EDTA is more accurate (with inulin being the gold standard), these measures are not routinely performed in clinical practice due to cumbersome and invasive operation. Instead, adjusting the dosage of drugs mainly excreted by the kidneys commonly relies on the levels of endogenous filtration markers such as serum creatinine (SCr) to measure GFR.^5,6^ In clinical administration, elevated SCr is often of great concern as drug eligibility and dosage depend on estimates of GFR. However, the correlation between an increase in SCr and a decrease in GFR is not absolute, thus failling to reflect deteriorating renal function or decreased drug excretion. For example, most patients with a GFR of about 40 mL/min appear to have normal CL_Cr_ (creatinine clearance).^7^ Besides, the SCr level may still be within the normal range on the first day of severe renal failure, and the measured GFR may not decrease significantly until 7-10 days.^8^ Furthermore, some drugs can reversibly increase SCr levels without affecting GFR.^9^ Therefore, it is recognized that SCr is an imperfect biomarker for evaluating GFR or adjusting drug dosage, which can be attributed to changes in creatinine biosynthesis, metabolism, renal tubular transport and drug interactions in most clinical settings.

 This review aims at systematizing the current knowledge on the factors that affect SCr levels *in vivo *and identifying the challenges of using creatinine as a biomarker for kidney function and measuring drug dosing adjustment.

## Factors affecting SCr level

###  Creatinine biosynthesis 

 Creatinine is mainly produced in skeletal muscles from the non-enzymatic dehydration and cyclization of creatine and phosphocreatine, and creatine is a nitrogenous organic acid produced by the liver, kidneys and pancreas,^10^ of which 75% is phosphorylated to produce phosphocreatine by creatine kinase (CK), while the remainder is present in its free form.^10,11^ The serum creatine level in adults is about 1.6-7.9 mg/L.^12^ A 70-kg man contains 120 g creatine, and roughly 1.7% of the total creatine pool (1.1% creatine/day and 2.6% phosphorylcreatine/day) is nonenzymatically converted to creatinine daily.^13,14^

**Figure 1:**
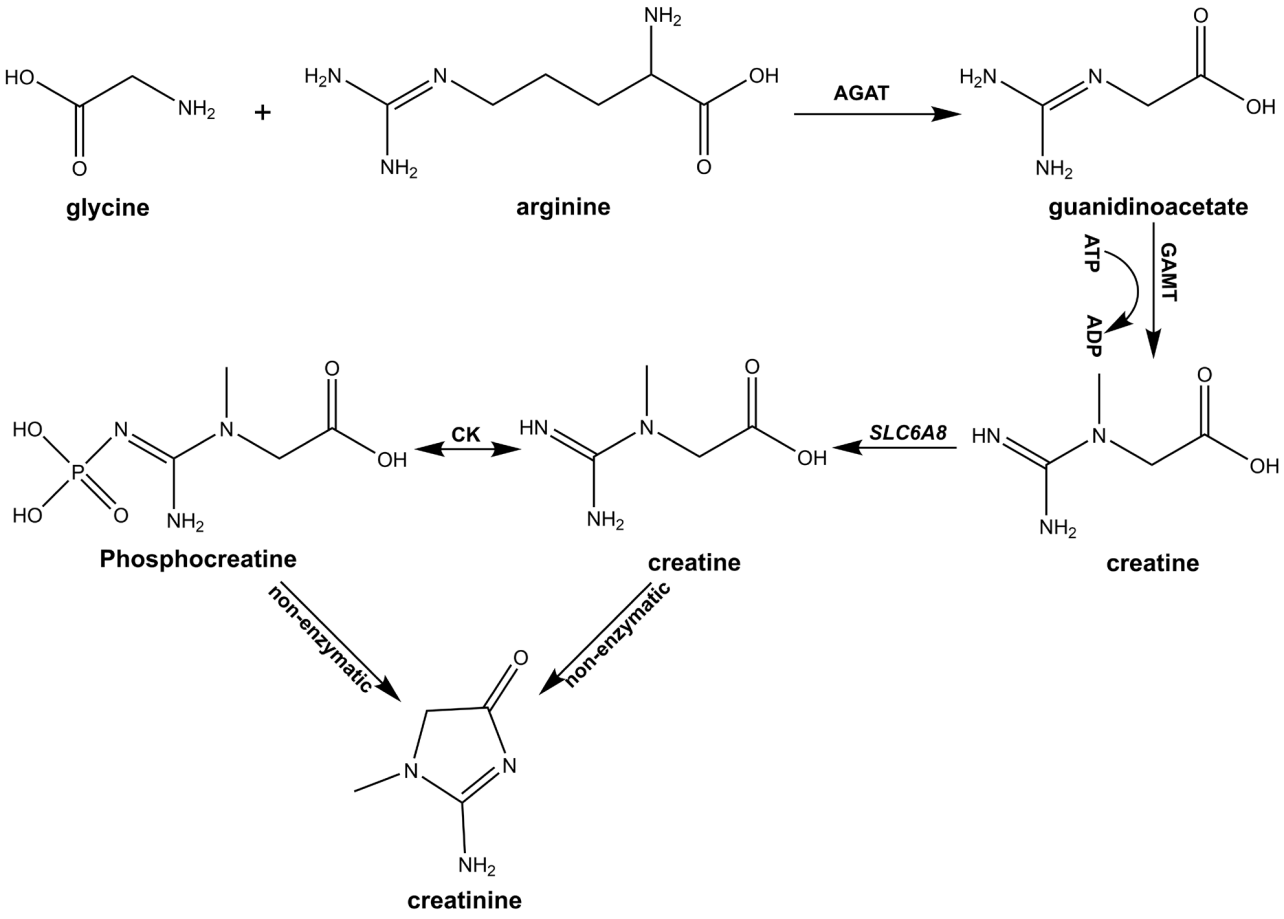
Creatinine biosynthesis. ADP, adenosine 5'-diphosphate; AGAT, L-arginine-glycine amidinotransferase; ATP, adenosine 5'-triphosphate; CK, creatine kinase; GAMT, guanidinoacetate methyltransferase; *SLC6A8*, solute carrier family 6 member 8

 As illustrated in Figure 1, the biosynthesis of endogenous creatinine is a multi-step process. The first step is to synthesize guanidine acetate in kidney catalyzed by L-arginine-glycine amidinotransferase (AGAT), mainly in the mitochondrial membrane space and less in cytoplasm. In the second step, guanidinoacetate methyltransferase (GAMT) facilitates the transfer of a methyl group from *S*-adenosylmethionine, producing creatine and *S-*adenosylhomocysteine in the liver. The third step is creatine transport via Na^+^-Cl^-^-dependent creatine transporter (*SLC6A8*), followed by CK-mediated creatine phosphorylation to form phosphocreatine. The final step is to form creatinine through non-enzymatic dehydration/cyclization of creatine, which can freely diffuse out of the cell and ultimately be removed in urine.

 Endogenous creatine synthesis is complicated due to the lack of specific enzymes required by most tissues, making dynamic interactions between metabolic enzymes and transportation between different tissues necessary.

 AGAT, the rate-limiting enzyme and *de novo* synthesis-initiating step, is predominantly expressed in the kidney. Despite the presence of significant amounts of AGAT in the livers of pigs, monkeys, and humans, it is widely acknowledged that the majority of guanidinoacetate synthesis predominantly occurs in the kidney.^15,16^ Creatine and L-ornithine exert negative pre-translational feedback on AGAT expression in the kidney.^17^ However, creatinine and phosphocreatine are both ineffective. AGAT expression may be under the control of hormonal factors, including estrogens, testosterone, thyroid hormones and growth hormone.^13,17,18^ In rats that have undergone thyroidectomy or hypophysectomy, AGAT activity in the kidney is reduced, but it can be restored by administering thyroxine or growth hormone, respectively. AGAT levels in rat kidneys are downregulated by estrogens and diethylstilbestrol, while upregulated by testosterone. Additionally, AGAT levels in kidneys, livers and other tissues are decreased in some situations, such as fasting, vitamin E deficiency and streptozotocin-induced diabetes.^19-21^

 GAMT, the second enzyme in creatine synthesis, is most strongly expressed in the liver, testis, caput epididymis and ovaries. As a whole, creatine synthesized by the liver is sufficient to meet the requirements for creatine in the entire body.^22^ Although the GAMT level in female liver is higher than that in males, estradiol, testosterone, cortisol, thyroxine and growth hormone have little effect on GAMT activity in rat liver.^23,24^ In contrast to the suppression of AGAT expression by creatine in the kidney, the expression of GAMT in the liver is not under the control of creatine or ornithine. The influencing factors and regulation of GATM are still unclear.

 Creatine transporter (*SLC6A8*) predominantly mediates the uptake of creatine rather than creatinine to skeletal muscle, brain, kidney and heart,^25^ and its expression and/or activity is regulated by diet, hormonal factors, guanidinoacetate and extracellular creatine concentration, with negative regulation by high creatine levels occurring more rapidly than the positive control mediated by creatine deficiency.^17,26^ Dietary creatine supplementation depresses the expression of the creatine transporter in rats.^27^ Importantly, dietary creatine supplementation results in a 3 to 20-fold increase in serum creatine concentration, but only a 10%-20% increase in muscle creatine.^17^ This result is attributed to the low permeability of creatine in muscles. Consistently, the creatine transporter expression is downregulated by extracellular creatine of > 0.1 μM (with IC50 ≈ 20-30 μM). More than 5 mM guanidinoacetate or guanidinopropionate also decreases creatine transport, but D-/L-ornithine, creatinine and phosphocreatine have no effect.^28^ Conversely, creatine transporter activity is inhibited by isoproterenol, norepinephrine, clenbuterol and *N*^6^,2′-O-dibutyryladenosine 3′,5′-cyclic monophosphate *in vitro*, which can be related to the regulation of intracellular cyclic adenosine monophosphate levels.^29^ In addition, the uptake of creatine is inhibited by the Na^+^-K^+^-ATPase inhibitors ouabain and digoxin. Insulin and insulin-like growth factor increase the activity of Na^+^-K^+^-ATPase, ultimately resulting in increased uptake of creatine.^30-32^

 CK is a central controller of cellular energy homeostasis, predominately located in skeletal muscles, myocardium and brain, and reversibly catalyzes the metabolism of creatine by utilizing ATP to generate phosphocreatine and ADP. Most tissues express two CK isoenzymes, dimeric cytosolic and octameric mitochondrial CK. Cytosolic CK consists of two subunits, B (brain type) or M (muscle type), which yields three isoenzymes: CK-MM, CK-BB and CK-MB.^33-35^ In addition to three cytosolic CK isoforms, there are two mitochondrial CK isoenzymes, the ubiquitous and sarcomeric forms.^33^ The presence of cytosolic and mitochondrial CK plays multiple roles in cellular energy homeostasis.^36-38^ In the healthy subject, total CK is mainly composed of the MM isoform, but depends on age, gender race, muscle mass as well as disease state (Supplementary Table S1).^39^

###  Creatinine metabolism 

 Creatinine is excreted exclusively through a combination of glomerular filtration and tubular secretion, with minimal binding to plasma proteins and negligible metabolism in healthy individuals. In severe renal insufficiency, up to 68% of generated creatinine may be metabolized or excreted via extrarenal routes.^40-42^ However, extrarenal elimination has not been observed in patients with mild to moderate renal insufficiency.^12^

**Figure 2:**
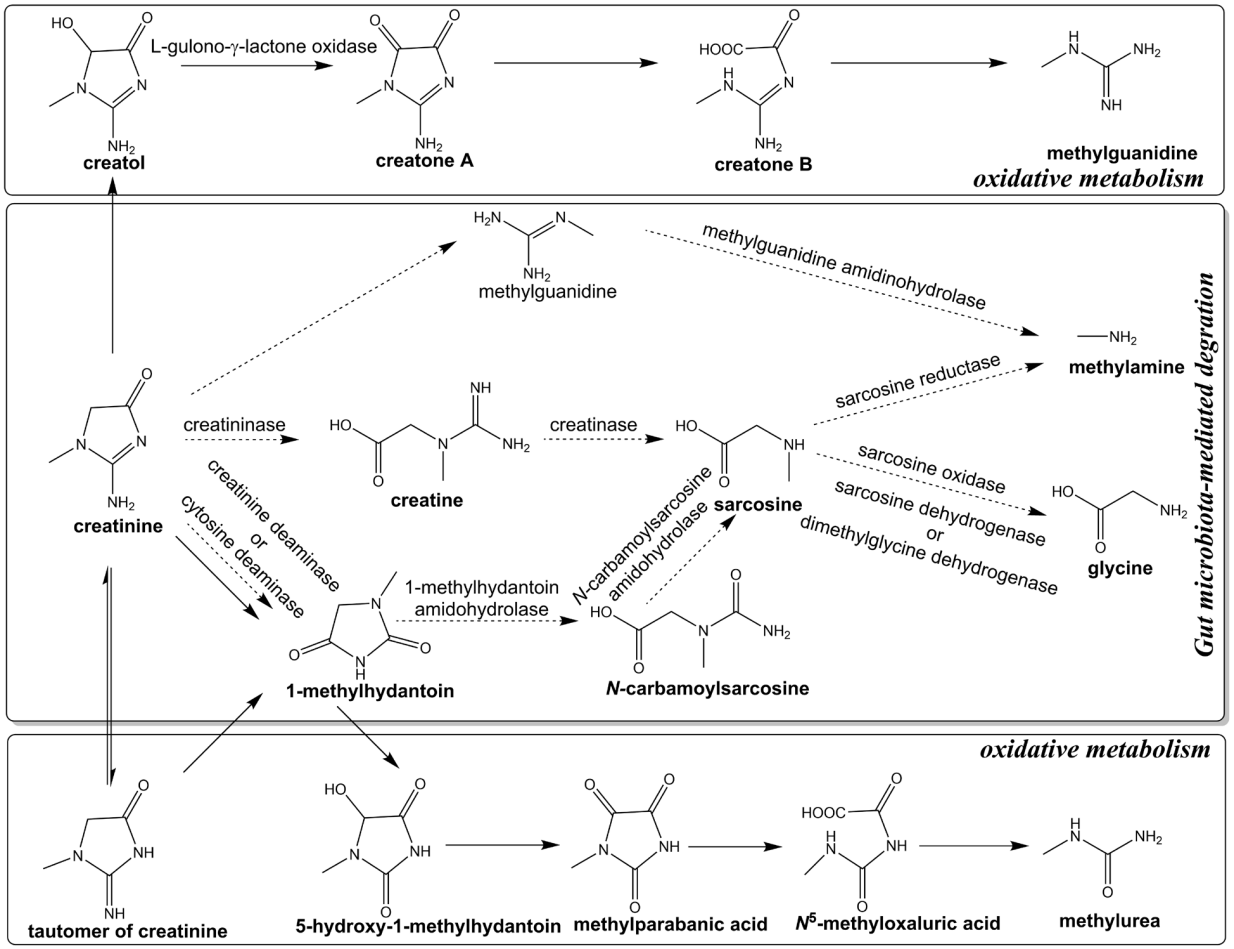
Creatinine metabolism

 Gut microbiota-mediated degradation and oxidative metabolism may facilitate the catabolism of creatinine (Figure 2).^17,43^ There may be two pathways of microbial-mediated degradation of creatinine: (1) Creatinine can be broken down into 1-methylhydantoin and ammonia through the action of creatinine deaminase and cytosine deaminase in various bacteria and fungi, and 1-methylhydantoin is further broken down into *N*-carbamoylsarcosine and sarcosine by 1-methylhydantoin amidohydrolase and *N*-carbamoylsarcosine amidohydrolase, respectively.^44,45^ In this pathway, 1-methylhydantoin amidohydrolase is a rate-limiting enzyme, and consequently, *N*-carbamoylsarcosine is in much lower concentration than other intermediary metabolites and even undetectable.^45^ (2) Creatinine is hydrolyzed to creatine which is partly reabsorbed or degraded by bacteria, and the production of creatine by creatininase is then degraded by creatinase to urea and sarcosine.^17,45^ Sarcosine is further converted to glycine by sarcosine oxidase or sarcosine dehydrogenase, and in the end to methylamine by sarcosine reductase. In addition, only a few studies have addressed the conversion of creatinine to methylguanidine, which can be further decomposed to methylamine via methylguanidine amidinohydrolase.^17,46,47^

 Two oxidative pathways of creatinine catabolism have been demonstrated: (1) Creatinine is metabolized to methylguanidine and the intermediate creatol, creatone A, or creatone B.^48,49^ However, it is unclear whether these steps of the pathway are enzyme-catalyzed reactions.^47,48,50,51^ ROS may selectively stimulate the formation of methylguanidine from creatinine.^52,53^ (2) Creatinine also can be converted to 1-methylhydantoin, which is further degraded to 5-hydroxy-1-methylhydantoin, methylparabanic acid, *N*^5^-methyloxaluric acid as well as the end product methylurea.^54,55^ As shown in Figure 2, the formation of 1-methylhydantoin from creatinine may depend on bacterial degradation rather than non-enzymatic metabolism.^17^ In patients with chronic renal failure (CRF) or uremia, the formation of creatinine degradation products is increased and may further deteriorate kidney function.^56,57^

###  Transport and excretion of creatinine

 The vectorial transport of cationic compounds, along with some anionic and zwitterionic compounds, is regulated by the organic cation transporter 2 (OCT2) located on the basolateral membrane and the multidrug and toxin extrusion proteins (MATE1 and MATE2-K) on the apical membrane. Many anionic drugs are transported by the uptake organic anion transporter 1 (OAT1), OAT2 and OAT3 on the basolateral membrane, as well as the efflux transporters multidrug resistance-associated protein (MRP) 2 and MRP4 on the apical membrane.^58^ Other transporters, such as organic anion transporting polypeptide 4C1 (OATP4C1), P-glycoprotein (P-gp), novel organic cation transporters (OCTN1 and OCTN2) and breast cancer resistance protein (BCRP), may also be involved in mediating the renal secretion of some compounds.^1^

 Renal tubular transporter-mediated uptake of creatinine via OCT2, OCT3, OAT1, OAT2, and OAT3 has been found in both *in vivo* and *in vitro* studies.^59-61^ Creatinine is a low affinity substrate for OCT2, with *in vitro K*_m_ values of 1.9 ± 0.4,^62^ 4.0 ± 0.3 mM^61^ or 56.4 ± 3.4 mM.^63^ However, both *K*_m_ values are significantly higher than the physiological (about 45-85 μM for male and 30-60 μM for female) and even the pathophysiological concentrations of creatinine in humans. Therefore, the function of hOCT2 is not saturated under physiological conditions. Single-nucleotide polymorphisms of OCT2 (rs2504954) have been associated with the SCr levels.^64^ The creatinine uptake mediated by OCT3 is similar to ^62,64^ or lower than that by OCT2,^61,63^ but the expression of renal OCT3 is extremely low *in vivo*. It is worth noting that in hyperuricemia rats, the plasma concentration of creatinine significantly increased, while its renal clearance decreased, and the renal clearance ratio of creatinine to inulin dropped from 1.62 to 1.09.^65^ Considering that the data were corrected for inulin clearance, this observation could be explained by a decrease in tubular secretion of OCT2 and/or MATE1 transporters, rather than a decrease of GFR.

 OAT1 and OAT3 are responsible for the uptake of many anionic compounds. Although the fact that creatinine at physiological pH is a foundation, the uptake of creatinine by mOAT1 (*K*_m_ = 6.7 mM) and mOAT3 (*K*_m_ > 10 mM) were observed *in vitro* and *in vivo.*^59,66^ However, several studies have demonstrated that creatinine is not a substrate for OAT1, aligning with findings that creatinine uptake is mediated by OAT3 rather than OAT1 or OAT2,^60-62,64^ but the contribution of OAT3 to creatinine clearance is significantly lower compared to that of OCT2.^63^ On the contrary, Ciarimboli et al found that creatinine was not transported by mOAT3 in cell lines transfected with mOAT3.^60,64^

 OAT2 is found in both the basolateral and apical membranes of human renal proximal tubules, whereas in rats, it is localized only in the apical membrane,^67^ and its mRNA level is 3-fold higher than that of OCT2.^68^ OAT2 has many substrates that are the same as OAT1 and OAT3. Creatinine is the substrate of OAT2 and has high affinity (*K*_m_ values of 0.80-0.99 mM),^62,67^ and the transport efficiency for OAT2 is approximately 37-1850 times that of OCT2, MATE1 and MATE2-K.^67^

 MATE1 and MATE2-K are responsible for the efflux of creatinine from renal tubular cells.^62,67,69^ Kinetic analyses demonstrated that creatinine has a low affinity for MATE1 and MATE2K, with *K*_m_ values of > 10 and > 20 mM, respectively.^67,70^ It is unclear whether MRP2, MRP4, P-gp and BCRP mediate renal tubular clearance of creatinine.

 It has been proven that creatinine can be reabsorbed in renal tubules (5%-10%), but its mechanism remains unclear.^63,67^ Researchers speculated that creatinine reabsorption could be mediated by OAT2^67^ or OAT4,^63^ which could also be a passive process during low urine flow.^71^

 There is still controversy surrounding renal tubular transporters mediated creatinine elimination.

 Our study demonstrated that the uptake of d3-creatinine was significantly enhanced in OCT2-overexpressing cells compared to control cells, but not MATE1, MATE2-K, OAT1, OAT2, OAT3, MRP4, OATP4C1, P-gp, PEPT2 and URAT1.^72^

###  Interactions between creatinine and drugs

 Early studies suggested that creatinine was mainly passively filtered at the glomerulus with little secretion or reabsorption in renal tubules, and impaired kidney function resulted in a reduction of CL_Cr_ accompanied by an elevation of SCr. However, several drugs have been reported to affect creatinine secretion in renal tubules, thereby causing a transient non-pathologic increase in SCr without altering GFR. These changes can be attributed to the reversible inhibition of transporters responsible for the tubular secretion of creatinine.^73^ It is thus an important issue to understand how an increase in SCr results from pathologic injury or reversibly inhibited secretion.

 To distinguish that an increase of SCr is due to inhibition of renal tubular transporters rather than pathological changes, Chu et al carried out a retrospective analysis of the effect of inhibition of renal tubular OCT2, MATE1 and MATE2-K on SCr levels based on in *vivo*-*vitro* correlations^74^ using a cutoff value of *C*_max_/IC50 > 0.1 and *C*_max_,_u_/IC50 > 0.1.^9^ The US Food and Drug Administration and the International Transporter Consortium recommend a cutoff value of *C*_max_/IC50 > 0.1 and *C*_max,u_/IC50 > 0.1 to evaluate the potential risk of drug-drug interactions (Table 1). They found that cimetidine,^75-78^ cobicistat,^62,79^ dolutegravir,^80,81^ dronedarone,^82^ 7-[(3R)-3-(1-aminocyclopropyl) pyrrolidin-1-yl]-1-[(1R,2S)-2-fluorocyclopropyl]-8-methoxy-4-oxoquinoline-3-carboxylic acid (DX-619),^83^ pyrimethamine,^84,85^ rilpivirine,^86-88^ ranolazine,^89^ ritonavir,^79,90^ salicylate,^91^ telaprevir,^92-94^ and trimethoprim^95-98^ reversibly increased SCr levels by ≥ 10% without affecting GFR, and amiodarone^99^ and vandetanib^100^ reversibly increased SCr levels by > 10% but changes in GFR were not observed. In the phase 1 study, INCB039110,^101,102^ an inhibitor of the Janus kinases (JAKs) with selectivity for JAK1, reversibly increased SCr but did not affect GFR.^101^ However, both *C*_max_/IC50 and *C*_max,u_/IC50 resulted in a false-negative prediction for telaprevir. In addition, ranitidine had a *C*_max,u_/IC50 higher than 0.1 for OCT2, MATE1 and MATE2-K, but had no effect on SCr or CL_Cr._.^60^

**Table 1:** Effect of compounds on SCr, CL_Cr_ and GFR in humans

**Compounds**	**Dose regimen**	**Increase of SCr (%)**	**Decrease of CL**_Cr_ **(%)**	**GFR**	***C***_max_ **(µM)**	***f***_u_	**Inhibited transporters#**
Amiodarone	400-200 mg or 400-400 mg, p.o., qid, 1 y	11	/	/	0.8–2.3	0.04	OCT2, MATE1, MATE2-K, P-gp
Cimetidine	400 mg, p.o, bid, 7 d 400 mg, p.o., qid, 3 wk 400-200-200-200 mg, p.o. 400-400-400-800 mg, p.o.	13.5 25.8 38.2 22.2	18.2 14.8, 37.5 35.5 20.3	NS NS NS NS	9.36 / / 18.7	0.80	OCT2, OAT2, OAT3, MATE1, MATE2-K
Cobicistat	150 mg, p.o., qd, 7 d	10.5	8	NS	2.21	0.08	OCT2, OAT2, MATE1, MATE2-K
Dolutegravir	50 mg, p.o., qd, 14 d 50 mg, p.o., bid, 14 d	9.1 16.7	10 14	NS NS	6.75 13.11	0.01	OCT2, MATE1, MATE2-K
Dronedarone	400 mg, p.o., bid, 7 d	10-15	13.8	NS	0.30	0.02	OCT2, MATE1, P-gp
DX-619	800 mg, i.v., qd, 4 d	32.3	27	NS	22.04	0.29-0.35	OCT2, MATE1, MATE2-K
Famotidine	10 mg, i.v., SD 20 mg, p.o., bid, 7 d 200 mg, p.o., SD	NS NS /	NS NS SI	NS / /	About 1.3 0.39 /	0.8	OCT1, OCT2, OCT3, MATE1, MATE2-K
INCB039110	600 mg, p.o., bid, 8 d	SI	/	NS	3	/	OCT2, OAT2, MATE1, MATE2-K
Pyrimethamine	50 mg, p.o., SD 100 mg, p.o., SD	SI 18.5	16.5, 20.0 /	NS NS	2.29 4.6	0.13	OCT2, MATE1, MATE2-K
Ranolazine	1000 mg, p.o., bid, 5 d	12.4	11 (NS)	NS	4.87	0.37	OCT2, MATE1, MATE2-K
Rilpivirine	25 mg, p.o., qd, 48 wk	small increase	/	NS	0.58	0.003	OCT2, MATE1, MATE2-K
Ritonavir	100 mg, p.o., qd, 7 d	NS	NS or 25	NS	2.16	0.015	OCT2, MATE1, MATE2-K, P-gp, OAT2, OATPs
Salicylate	4 g/d, p.o., 10 d	38.4	24.7	NS	/	/	OAT1
Telaprevir	750 mg, p.o., tid, 12 wk	SI	/	NS	5.82	0.04-0.24	P-gp, but not OCT2 and MATE1/2-K
Trimethoprim	5 mg/kg, p.o., bid, 10 d 5 mg/kg, p.o., qid, 10 d 100 mg, p.o., bid, 10 d 200 mg, p.o., bid, 14 d	22.2 31.3 14.8 18.4	21.3 16.0 / 21.8	/ / NS NS	17.5 29.6 / 9.92	0.58	OCT2, MATE1, MATE2-K
Vandetanib	300 mg, p.o., qd, SD	SI	/	/	0.25-0.27	0.10	OCT2, MATE1, MATE2-K

/, data are not reported or available; bid, twice daily; *C*_max_, maximum plasma concentration; CL_Cr_, creatinine clearance; d, day; DX-619, 7-[(3R)-3-(1-aminocyclopropyl)pyrrolidin-1-yl]-1-[(1R,2S)-2-fluorocyclopropyl]-8-methoxy-4-oxoquinoline-3-carboxylic acid; *f*_u_, plasma unbound fraction; GFR, glomerular filtration rate; INCB039110, (2-(3-(4-(7H-pyrrolo[2,3-day]pyrimidin-4-yl)-1H-pyrazol-1-yl)-1-(1-(3-fluoro-2-(trifluoromethyl)isonicotinoyl)piperidin- 4-yl)azetidin-3-yl)acetonitrile); i.v., intravenous; MATE, multidrug and toxin extrusion protein; NS, no significance; OAT, organic anion transporter; OATPs, organic anion transporting polypeptides; OCT2, Organic cation transporter 2; P-gp, P-glycoprotein; p.o., oral; qd, once daily, qid, four times daily; SCr, serum creatinine; SI, significantly increased compared with baseline level; tid, three times daily; ^#^ Data from http://transportal.compbio.ucsf.edu.

 Eisner et al demonstrated that* para*-aminohippuric acid, a classical substrate of OAT1, induced a decrease in creatinine secretion and increased SCr levels.^59^ Notably, tubular handling of creatinine could be dependent on serum albumin levels.^103^ Collectively, the increase of SCr or decrease of CL_Cr_ can be attributed to the inhibition of creatinine secretion mediated by one or more renal tubular transporters. However, inhibition of renal tubular transporters does not necessarily lead to elevated SCr.

## Challenges of creatinine as a biomarker for renal function and drug dosing adjustment

**Figure 3:**
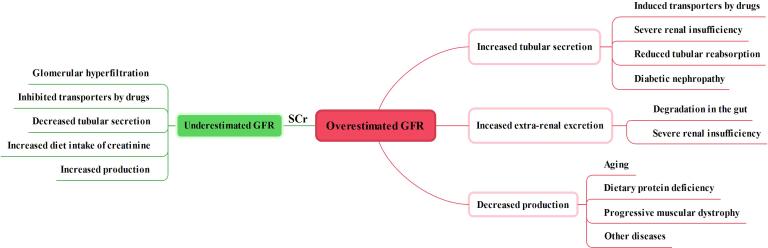
Influencing factors of SCr in evaluating GFR. GFR, glomerular filtration rate; SCr, serum creatinine

 There is indeed a relationship between GFR and CL_Cr_ in young adults without renal diseases.^8^ However, SCr is an imperfect biomarker for estimating GFR and its levels can be influenced by various factors mentioned above. Firstly, as fractional secretion varies inversely with GFR, SCr levels cannot be changed by renal tubular hypersecretion of creatinine with the deterioration of glomerular function.^7^ Secondly, some drugs act by competitively inhibiting the transport of creatinine in renal tubules as a result of SCr elevation without changing GFR. Thirdly, a substantial fraction of creatinine is metabolized rather than excreted with a sharply decreased GFR. Fourthly, the rise in SCr following a reduction in GFR is delayed due to kinetic changes in creatinine production and accumulation. For example, the serum half-life of creatinine is approximately 4 h at a normal GFR of 120 mL/min/1.73 m^2^ but extends to 16 hours at a GFR of 30 mL/min/1.73 m^2^.^9^ Fifthly, SCr is also affected by other factors, including weight, gender, age, muscle metabolism as well as intake or use of protein supplements. Notably, glomerular hyperfiltration occurring as a consequence of underlying disease is often ignored because of no change or mild decrease in SCr.^104-107^ Therefore, appropriate increases in drug dosing would rarely be carried out, which would lead to subtherapeutic concentrations of drugs^108^ (Figure 3).

 Variations in creatine pool size can substantially impact creatinine production. Total muscle mass is a critical factor in determining creatine pool size, and conditions such as aging,^109^ dietary protein deficiency, progressive muscular dystrophy,^110^ chronic glucocorticoid therapy,^111^ sepsis,^112^ hyperthyroidism and poliomyelitis,^113^ can decrease the production of creatinine. The size of the creatine pool is diminished during a creatine-free period or dietary protein deficiency, but the rate of conversion of creatine to creatinine remains unaffected.^114,115^ Although creatinine levels in meat (0.2-0.4 mg creatinine and 3.5-5 mg creatine per gram of uncooked lean beef) are very low, meat is also a major source of creatinine as a consequence of high conversion ratio from creatine to creatinine (18%-65%).^12,116,117^ Consequently, the excretion of creatinine decreases by 10-30% when reducing dietary meat content. Moreover, a slight change in the turnover ratio of creatine will have a significant impact on creatinine production because of the relatively large pool size of the creatine. Fitch and Sinton found that the turnover ratio of creatine increased to 2.2%-3.8% per day in some patients with muscular dystrophy.^118^

 Tubular secretion of creatinine was identified in an early study investigating the clearance of exogenously administered creatinine.^119^ The exogenous creatinine excretion was decreased in a high plasma creatinine state produced by infusion of creatinine, which could be related to the competitive inhibition of renal tubular secretion of creatinine.^119^ As discussed above, some compounds can increase SCr by up to 40% without altering GFR.^83,91^ During severe renal insufficiency the elimination of creatinine via glomerular filtration decreases and tubular secretion is increased by as much as 60%.^7,120^ Thus, the contribution of active secretion of creatinine in renal tubules could result in an overestimation of GFR.

 Creatinine is eliminated solely by the kidney in healthy people. Extrarenal creatinine elimination occurs only in patients with severe renal insufficiency. This mechanism is thought to result from the degradation of creatinine in the intestinal lumen by gut microbiota. The increased level of creatinine caused by renal dysfunction induces bacterial creatininase activity, resulting in degradation and loss of creatinine,^42,121^ and creatinine degradation can be abolished by antibiotics.^121^ Consequently, the GFR could be overestimated by CL_Cr_ as a result of extrarenal elimination.

 Creatinine synthesis, metabolism and elimination are altered in certain disease states, which could lead to inaccurate assessment in GFR by using SCr clearance. Aging is linked to changes in renal structure and function, with GFR decreasing by approximately 8-10 mL/min/1.73 m^2^ per decade after the age of 30.^122,123^ Consistently, renal clearance of creatinine is also decreased with aging. However, this fall in CL_Cr_ with the progressive decrease in GFR is commonly accompanied by a decrease in creatinine production, and consequently, SCr may not be affected.^124^

 GFR in early pregnancy increases by 50% compared to that of later pregnancy levels.^125^ However, the ratio of creatinine to inulin clearance is slightly above 1.0 (normal ranges from 1.1 to 1.4) in the first and early second trimester, and is approximate or slightly lower than that in later pregnancy, suggesting that tubular secretion of creatinine is attenuated during pregnancy, especially in the latter half. As a result of the decrease in CL_Cr_ in pregnancy, a SCr concentration above 8 mg/L is an abnormal result.^126^

 Acute kidney injury (AKI) leads to a rapid decrease in GFR. Although GFR is effectively equal to zero at the early stage of AKI, SCr may be only slightly above baseline. Conversely, SCr continues to increase at the early stage of recovery from AKI.^127^ In patients with CRF, because of increased tubular secretion of creatinine, the creatinine/inulin clearance ratio is as high as 2.5 at a lower GFR.^120^ Of note, tubular clearance of creatinine is significantly enhanced at GFR from 40 to 80 mL/min/1.73 m^2^.^7^ Even if the GFR is reduced to 15 mL/min/1.73 m^2^, SCr changed by only 2.0 mg/L, but these changes could not be considered significant.^12^ In addition, reduced creatinine production and increased extrarenal metabolism are also observed in CRF.^40,114^ Thus, the rate of decline in SCr may not accurately reflect the rate of decline in GFR in some instances of physiological and pathological changes, which can result in incorrect drug dosages.

 Diabetes mellitus is often associated with a deterioration in kidney function.^128^ Some studies found that GFR increased by 27% and 16% in recently diagnosed patients with T1DM^107,129^ and T2DM,^130^ respectively. Generally, GFR in untreated diabetes is higher than that in short-term insulin-treated diabetes.^131^ Consistently, CL_Cr_ is increased in early diabetes. During diabetic ketoacidosis and diabetic coma, GFR decreases and SCr increases. However, the decline in GFR is not associated with a parallel increase in SCr. McCance and Widdowson found three of four patients with diabetic coma had the creatinine/inulin clearance ratio less than 1 (0.42-0.85),^132^ suggesting that creatinine could also undergo the reabsorption in renal tubules. In diabetic nephropathy, SCr levels remain within the normal range despite the GFR is as low as 36 mL/min/1.73 m^2^,^133^ which could be attributed to enhanced secretion of creatinine in renal tubules. Consequently, changes in SCr do not reliably predict variations in GFR.

## Summary and Perspective

 A reliable assessment of renal function is essential for evaluating renal disease stage and progression, determining the need for dialysis therapy, screening kidney donors and adjusting drug dosages. GFR is generally accepted as the best overall measure of kidney function. Over 70 equations based on SCr levels have been developed to estimate GFR. Among these, the Cockcroft-Gault formula and the Modification of Diet in Renal Disease (MDRD) formula are the most extensively studied and widely applied.^2,134,135^ Over the years, the importance of SCr determination in diagnosing renal disease and monitoring disease progression cannot be overemphasized. However, a large number of researchers have pointed out that there is no absolute correlation between GFR and SCr.^136-139^ The relationship between SCr and measured GFR is not linear but curvilinear, and a given value of SCr can be associated with a wide range of measured GFR values (30-90 mL/min/1.73 m^2^),^137^ which can cause difficulty in distinguishing between a normal GFR and an abnormal one.^140^ The estimated GFR by SCr is insensitive at a GFR above 60 ml/min/1.73 m^2^, creating a “creatinine-blind range”,^138,141^ and thus the measurement of SCr is limited as a diagnostic marker for the early stages of renal injury.^142^ As a result, SCr as a marker for adjusting drug dosages may not achieve satisfactory therapeutic objectives,^143^ which can be attributed to failure to recognize the variations in non-GFR determinants including generation, tubular secretion or reabsorption and extra-renal elimination of creatinine. To accurately predict kidney function via SCr levels, the factors affecting creatinine synthesis, metabolism and elimination would need to be fully considered in clinical settings. Under creatinine intake control, simultaneous monitoring of plasma levels of creatinine and its precursors, guanidinoacetate and creatine, can indirectly reflect creatinine synthesis. Although it is difficult to evaluate creatinine metabolism mediated by gut microbiota *in vivo*, renal or extra-renal elimination of creatinine can be determined via ECT/PET imaging using radioactively labeled creatinine. In view of the unclear mechanism of renal tubular transport of creatinine, it is particularly important to elucidate the renal tubular transporters that mediate elimination of creatinine.

 Some researchers have argued that serum cystatin C is a better biomarker for estimating GFR than SCr.^144,145^ However, serum concentration of cystatin C can be affected by inflammation and changes in protein catabolism,^146,147^ and the biological variation in cystatin C levels is far higher than that in creatinine.^138^ One study published in the *New England Journal of Medicine* demonstrated that the estimated GFR by serum cystatin C was not more accurate than SCr, and the combination of SCr and serum cystatin C was more precise than equations using either marker individually for estimating GFR.^148^ In addition, some investigators suggested that cystatin C at higher levels of GFR might be a better filtration marker than creatinine.^149,150^ Thus, to some extent, the use of cystatin C can avoid the risk associated with the “creatinine-blind range”, and estimating GFR by the combination of serum cystatin C and SCr may be a better choice.

 Kidney tubular secretion is another important renal functional parameter and 61% of all drugs are eliminated through tubular secretion mediated by transporters rather than through glomerular filtration.^146^ Thus, a strategy of drug dosing adjustment should be based on the actual mechanism of kidney drug elimination, not just on the GFR. Importantly, renal tubules are vulnerable to a variety of injuries.^151^ Based on these reasons, the development of markers for renal tubular transporters will be of great use in the early diagnosis of renal injury and adjustment of drug dosages. In recent years, growing research has focused on identifying potential biomarkers for renal tubular transporters, with several endogenous compounds being recognized as biomarker of these transporters. Thiamine and N-methylnicotinamide are potential substrates for the cation transport system (OCT2-MATE1/2-K) in renal tubules.^69,152-154^ Hippurate and taurine, cyclic guanosine monophosphate, and 6β-hydroxycortisol and glycochenodeoxycholate sulfate have been proposed as endogenous probes for the evaluation of OAT1, OAT2 and OAT3 function, respectively.^155-157^ In addition, some tubular proteins, neutrophil gelatinase-associated lipocalin, kidney injury molecule-1 and N-acetyl-β-D-glucosaminidase have all emerged as early and sensitive markers for renal tubular injury.^158^ Unfortunately, these markers are not currently used to adjust drug dosages clinically. Therefore, the evaluation system of renal excretion pathways of drugs based on multiple biomarkers should be established.

**Figure 4:**
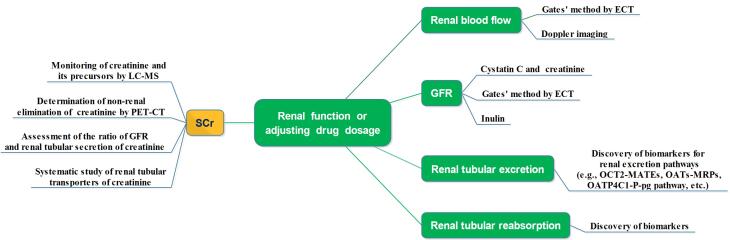
Kidney function evaluation and drug dosing adjustment from the perspective of SCr and new approaches. ECT, emission computed tomography; GFR, glomerular filtration rate; LC-MS, liquid chromatography-tandem mass spectrometry, MATE, multidrug and toxin extrusion protein; OAT, organic anion transporter; OATP4C1, organic anion transporting polypeptide 4C1; OCT2, Organic cation transporter 2; P-gp, P-glycoprotein; PET-CT, positron-emission tomography computed tomography

 Renal elimination of endogenous and exogenous compounds is affected by many factors, including renal blood flow, GFR, and renal tubular excretion and reabsorption, and monitoring these changes will be conducive to evaluating renal excretory function. When creatinine is used as a marker for GFR and drug dosing adjustment, changes in its synthesis, metabolism and excretion and other influencing factors need to be fully considered (Figure 4).

## Conclusion

 SCr as a biomarker for evaluating GFR and adjusting the dosage of drugs is imperfect, which is particularly reflected in low correlation, insensitivity and high variation of non-GFR determinants. This could be related to changes in the generation, tubular secretion or reabsorption, and extra-renal elimination of creatinine. However, there is a lack of latest research evidence about the biosynthesis, metabolism and extra-renal elimination of creatinine. Therefore, in order to better evaluate renal function and adjust drug dosages, studies on the elimination pathways of creatinine *in vivo* should be necessary, and the combination of multiple markers of renal function should be developed.

## Competing Interests

 The authors declare no conflict of interest.

## Ethical Approval

 Not applicable.

## Supplementary Files

Table S1. Enzymes and transporters in creatinine biosynthesis

## References

[1] Morrissey KM, Stocker SL, Wittwer MB, Xu L, Giacomini KM (2013). Renal transporters in drug development. Annu Rev PharmacolToxicol.

[2] Stevens LA, Coresh J, Greene T, Levey AS (2006). Assessing kidney function--measured and estimated glomerular filtration rate. N Engl J Med.

[3] Delanghe JR, Speeckaert MM (2011). Creatinine determination according to Jaffe-what does it stand for?. NDT Plus.

[4] Rehberg PB (1926). Studies on kidney function: the rate of filtration and reabsorption in the human kidney. Biochem J.

[5] Sunder S, Jayaraman R, Mahapatra HS, Sathi S, Ramanan V, Kanchi P (2014). Estimation of renal function in the intensive care unit: the covert concepts brought to light. J Intensive Care.

[6] Li DY, Yin WJ, Yi YH, Zhang BK, Zhao J, Zhu CN (2019). Development and validation of a more accurate estimating equation for glomerular filtration rate in a Chinese population. Kidney Int.

[7] Shemesh O, Golbetz H, Kriss JP, Myers BD (1985). Limitations of creatinine as a filtration marker in glomerulopathic patients. Kidney Int.

[8] Spencer K (1986). Analytical reviews in clinical biochemistry: the estimation of creatinine. Ann Clin Biochem.

[9] Chu X, Bleasby K, Chan GH, Nunes I, Evers R (2016). Transporters affecting biochemical test results: creatinine-drug interactions. Clin PharmacolTher.

[10] Heymsfield SB, Arteaga C, McManus C, Smith J, Moffitt S (1983). Measurement of muscle mass in humans: validity of the 24-hour urinary creatinine method. Am J Clin Nutr.

[11] Ellery SJ, Walker DW, Dickinson H (2016). Creatine for women: a review of the relationship between creatine and the reproductive cycle and female-specific benefits of creatine therapy. Amino Acids.

[12] Perrone RD, Madias NE, Levey AS (1992). Serum creatinine as an index of renal function: new insights into old concepts. Clin Chem.

[13] Walker JB (1979). Creatine: biosynthesis, regulation, and function. Adv EnzymolRelat Areas Mol Biol.

[14] Wyss M, Schulze A (2002). Health implications of creatine: can oral creatine supplementation protect against neurological and atherosclerotic disease?. Neuroscience.

[15] Goldman R, Moss JX (1960). Creatine synthesis after creatinine loading and after nephrectomy. Proc Soc Exp Biol Med.

[16] Edison EE, Brosnan ME, Meyer C, Brosnan JT (2007). Creatine synthesis: production of guanidinoacetate by the rat and human kidney in vivo. Am J Physiol Renal Physiol.

[17] Wyss M, Kaddurah-Daouk R (2000). Creatine and creatinine metabolism. Physiol Rev.

[18] Van Pilsum JF, McGuire DM, Towle H (1982). On the mechanism of the alterations of rat kidney transamidinase activities by diet and hormones. Adv Exp Med Biol.

[19] Funahashi M, Kato H, Shiosaka S, Nakagawa H (1981). Formation of arginine and guanidinoacetic acid in the kidney in vivo Their relations with the liver and their regulation. J Biochem.

[20] Fitch CD, Hsu C, Dinning JS (1961). The mechanism of kidney transamidinase reduction in vitamin E-deficient rabbits. J Biol Chem.

[21] Kim GS, Chevli KD, Fitch CD (1983). Fasting modulates creatine entry into skeletal muscle in the mouse. Experientia.

[22] Xue GP, Snoswell AM, Fishlock RC (1988). Quantitative study on creatine metabolism in sheep tissues. Biochem Int.

[23] Lee H, Ogawa H, Fujioka M, Gerton GL (1994). Guanidinoacetate methyltransferase in the mouse: extensive expression in Sertoli cells of testis and in microvilli of caput epididymis. Biol Reprod.

[24] Carlson M, Van Pilsum JF (1973). S-adenosylmethionine: guanidinoacetate N-methyltransferase activities in livers from rats with hormonal deficiencies or excesses. Proc Soc Exp Biol Med.

[25] Snow RJ, Murphy RM (2001). Creatine and the creatine transporter: a review. Mol Cell Biochem.

[26] Joncquel-Chevalier Curt M, Voicu PM, Fontaine M, Dessein AF, Porchet N, Mention-Mulliez K (2015). Creatine biosynthesis and transport in health and disease. Biochimie.

[27] Guerrero-Ontiveros ML, Wallimann T (1998). Creatine supplementation in health and disease Effects of chronic creatine ingestion in vivo: down-regulation of the expression of creatine transporter isoforms in skeletal muscle. Mol Cell Biochem.

[28] Loike JD, Zalutsky DL, Kaback E, Miranda AF, Silverstein SC (1988). Extracellular creatine regulates creatine transport in rat and human muscle cells. Proc Natl Acad Sci U S A.

[29] Odoom JE, Kemp GJ, Radda GK (1996). The regulation of total creatine content in a myoblast cell line. Mol Cell Biochem.

[30] Bennett SE, Bevington A, Walls J (1994). Regulation of intracellular creatine in erythrocytes and myoblasts: influence of uraemia and inhibition of Na,K-ATPase. Cell BiochemFunct.

[31] Gonda O, Quastel JH (1962). Effects of ouabain on cerebral metabolism and transport mechanisms in vitro. Biochem J.

[32] Steenge GR, Lambourne J, Casey A, Macdonald IA, Greenhaff PL (1998). Stimulatory effect of insulin on creatine accumulation in human skeletal muscle. Am J Physiol.

[33] Schlattner U, Forstner M, Eder M, Stachowiak O, Fritz-Wolf K, Wallimann T (1998). Functional aspects of the X-ray structure of mitochondrial creatine kinase: a molecular physiology approach. Mol Cell Biochem.

[34] Eppenberger ME, Eppenberger HM, Kaplan NO (1967). Evolution of creatine kinase. Nature.

[35] Wallimann T, Wyss M, Brdiczka D, Nicolay K, Eppenberger HM (1992). Intracellular compartmentation, structure and function of creatine kinase isoenzymes in tissues with high and fluctuating energy demands: the ‘phosphocreatine circuit’ for cellular energy homeostasis. Biochem J.

[36] Dzeja PP, Terzic A (2003). Phosphotransfer networks and cellular energetics. J Exp Biol.

[37] Saks VA, Ventura-Clapier R, Aliev MK (1996). Metabolic control and metabolic capacity: two aspects of creatine kinase functioning in the cells. BiochimBiophys Acta.

[38] Schlattner U, Tokarska-Schlattner M, Wallimann T (2006). Mitochondrial creatine kinase in human health and disease. BiochimBiophys Acta.

[39] Brancaccio P, Maffulli N, Limongelli FM (2007). Creatine kinase monitoring in sport medicine. Br Med Bull.

[40] Mitch WE, Collier VU, Walser M (1980). Creatinine metabolism in chronic renal failure. Clin Sci (Lond).

[41] Levey AS, Perrone RD, Madias NE (1988). Serum creatinine and renal function. Annu Rev Med.

[42] Jones JD, Burnett PC (1974). Creatinine metabolism in humans with decreased renal function: creatinine deficit. Clin Chem.

[43] Owens CW, Albuquerque ZP, Tomlinson GM (1979). In vitro metabolism of creatinine, methylamine and amino acids by intestinal contents of normal and uraemic subjects. Gut.

[44] Kim JM, Shimizu S, Yamada H (1987). Cytosine deaminase that hydrolyzes creatinine to N-methylhydantoin in various cytosine deaminase-forming microorganisms. Arch Microbiol.

[45] Shimizu S, Kim JM, Shinmen Y, Yamada H (1986). Evaluation of two alternative metabolic pathways for creatinine degradation in microorganisms. Arch Microbiol.

[46] Nakajima M, Shirokane Y, Mizusawa K (1980). A new amidinohydrolase, methylguanidine amidinohydrolase from Alcaligenes sp N-42. FEBS Lett.

[47] Jones JD, Burnett PC (1972). Implication of creatinine and gut flora in the uremic syndrome: induction of “creatininase” in colon contents of the rat by dietary creatinine. Clin Chem.

[48] Fujitsuka N, Yokozawa T, Oura H, Akao T, Kobashi K, Ienaga K (1993). L-gulono-gamma-lactone oxidase is the enzyme responsible for the production of methylguanidine in the rat liver. Nephron.

[49] Nakamura K, Ienaga K, Nakano K, Nakai M, Nakamura Y, Hasegawa G (1994). Creatol, a creatinine metabolite, as a useful determinant of renal function. Nephron.

[50] Gonella M, Barsotti G, Lupetti S, Giovannetti S (1975). Factors affecting the metabolic production of methylguanidine. Clin Sci Mol Med.

[51] Nakamura K, Ienaga K (1990). Creatol (5-hydroxycreatinine), a new toxin candidate in uremic patients. Experientia.

[52] Aoyagi K, Akiyama K, Kuzure Y, Takemura K, Nagase S, Ienaga K (1998). Synthesis of creatol, a hydroxyl radical adduct of creatinine and its increase by puromycin aminonucleoside in isolated rat hepatocytes. Free Radic Res.

[53] Yokozawa T, Chung HY, Dong E, Oura H (1995). Confirmation that magnesium lithospermate B has a hydroxyl radical-scavenging action. Exp ToxicolPathol.

[54] Ienaga K, Nakamura K, Yamakawa M, Toyomaki Y, Matsuura H, Yokozawa T, et al. The use of 13C-labelling to prove that creatinine is oxidized by mammals into creatol and 5-hydroxy-1-methylhydantoin. J Chem Soc Chem Commun 1991(7):509-10. 10.1039/c39910000509.

[55] Ienaga K, Nakamura K, Naka F, Goto T (1988). The metabolism of 1-methylhydantoin via 5-hydroxy-1-methylhydantoin in mammals. BiochimBiophys Acta.

[56] Orita Y, Ando A, Tsubakihara Y, Mikami H, Kikuchi T, Nakata K (1981). Tissue and blood cell concentration of methylguanidine in rats and patients with chronic renal failure. Nephron.

[57] Yokozawa T, Mo ZL, Oura H (1989). Comparison of toxic effects of methylguanidine, guanidinosuccinic acid and creatinine in rats with adenine-induced chronic renal failure. Nephron.

[58] Burckhardt G, Wolff NA (2000). Structure of renal organic anion and cation transporters. Am J Physiol Renal Physiol.

[59] Eisner C, Faulhaber-Walter R, Wang Y, Leelahavanichkul A, Yuen PS, Mizel D (2010). Major contribution of tubular secretion to creatinine clearance in mice. Kidney Int.

[60] Mathialagan S, Rodrigues AD, Feng B (2017). Evaluation of renal transporter inhibition using creatinine as a substrate in vitro to assess the clinical risk of elevated serum creatinine. J Pharm Sci.

[61] Urakami Y, Kimura N, Okuda M, Inui K (2004). Creatinine transport by basolateral organic cation transporter hOCT2 in the human kidney. Pharm Res.

[62] Lepist EI, Zhang X, Hao J, Huang J, Kosaka A, Birkus G (2014). Contribution of the organic anion transporter OAT2 to the renal active tubular secretion of creatinine and mechanism for serum creatinine elevations caused by cobicistat. Kidney Int.

[63] Imamura Y, Murayama N, Okudaira N, Kurihara A, Okazaki O, Izumi T (2011). Prediction of fluoroquinolone-induced elevation in serum creatinine levels: a case of drug-endogenous substance interaction involving the inhibition of renal secretion. Clin PharmacolTher.

[64] Ciarimboli G, Lancaster CS, Schlatter E, Franke RM, Sprowl JA, Pavenstädt H (2012). Proximal tubular secretion of creatinine by organic cation transporter OCT2 in cancer patients. Clin Cancer Res.

[65] Nishizawa K, Yoda N, Morokado F, Komori H, Nakanishi T, Tamai I (2019). Changes of drug pharmacokinetics mediated by downregulation of kidney organic cation transporters MATE1 and OCT2 in a rat model of hyperuricemia. PLoS One.

[66] Vallon V, Eraly SA, Rao SR, Gerasimova M, Rose M, Nagle M (2012). A role for the organic anion transporter OAT3 in renal creatinine secretion in mice. Am J Physiol Renal Physiol.

[67] Shen H, Liu T, Morse BL, Zhao Y, Zhang Y, Qiu X (2015). Characterization of organic anion transporter 2 (SLC22A7): a highly efficient transporter for creatinine and species-dependent renal tubular expression. Drug MetabDispos.

[68] Cheng Y, Vapurcuyan A, Shahidullah M, Aleksunes LM, Pelis RM (2012). Expression of organic anion transporter 2 in the human kidney and its potential role in the tubular secretion of guanine-containing antiviral drugs. Drug MetabDispos.

[69] Tanihara Y, Masuda S, Sato T, Katsura T, Ogawa O, Inui K (2007). Substrate specificity of MATE1 and MATE2-K, human multidrug and toxin extrusions/H( + )-organic cation antiporters. BiochemPharmacol.

[70] Tsuda M, Terada T, Mizuno T, Katsura T, Shimakura J, Inui K (2009). Targeted disruption of the multidrug and toxin extrusion 1 (MATE1) gene in mice reduces renal secretion of metformin. Mol Pharmacol.

[71] Chesley LC (1938). Renal excretion at low urine volumes and the mechanism of oliguria. J Clin Invest.

[72] Ma Y, Zhang M, Yang J, Zhu L, Dai J, Wu X (2023). Characterization of the renal tubular transport of creatinine by activity-based protein profiling and transport kinetics. Eur J Pharm Sci.

[73] Casado JL, Monsalvo M, Vizcarra P, Fontecha M, Serrano-Villar S, Moreno S (2019). Evaluation of kidney function in HIV-infected patients receiving an antiretroviral regimen containing one or two inhibitors of the tubular secretion of creatinine. HIV Med.

[74] Chu X, Bleasby K, Chan GH, Nunes I, Evers R (2016). The complexities of interpreting reversible elevated serum creatinine levels in drug development: does a correlation with inhibition of renal transporters exist?. Drug MetabDispos.

[75] Dutt MK, Moody P, Northfield TC (1981). Effect of cimetidine on renal function in man. Br J Clin Pharmacol.

[76] Hilbrands LB, Artz MA, Wetzels JF, Koene RA (1991). Cimetidine improves the reliability of creatinine as a marker of glomerular filtration. Kidney Int.

[77] Ishigami M, Sezai Y, Shimada Y, Maeda T, Yabuki S (1989). Effects of famotidine, a new histamine H2-receptor antagonist, on renal function. Nihon Jinzo Gakkai Shi.

[78] Sansoè G, Ferrari A, Castellana CN, Bonardi L, Villa E, Manenti F (2002). Cimetidine administration and tubular creatinine secretion in patients with compensated cirrhosis. Clin Sci (Lond).

[79] German P, Liu HC, Szwarcberg J, Hepner M, Andrews J, Kearney BP (2012). Effect of cobicistat on glomerular filtration rate in subjects with normal and impaired renal function. J Acquir Immune DeficSyndr.

[80] Koteff J, Borland J, Chen S, Song I, Peppercorn A, Koshiba T (2013). A phase 1 study to evaluate the effect of dolutegravir on renal function via measurement of iohexol and para-aminohippurate clearance in healthy subjects. Br J Clin Pharmacol.

[81] Weller S, Borland J, Chen S, Johnson M, Savina P, Wynne B (2014). Pharmacokinetics of dolutegravir in HIV-seronegative subjects with severe renal impairment. Eur J Clin Pharmacol.

[82] Tschuppert Y, Buclin T, Rothuizen LE, Decosterd LA, Galleyrand J, Gaud C (2007). Effect of dronedarone on renal function in healthy subjects. Br J Clin Pharmacol.

[83] Sarapa N, Wickremasingha P, Ge N, Weitzman R, Fuellhart M, Yen C (2007). Lack of effect of DX-619, a novel des-fluoro(6)-quinolone, on glomerular filtration rate measured by serum clearance of cold iohexol. Antimicrob Agents Chemother.

[84] Opravil M, Keusch G, Lüthy R (1993). Pyrimethamine inhibits renal secretion of creatinine. Antimicrob Agents Chemother.

[85] Kusuhara H, Ito S, Kumagai Y, Jiang M, Shiroshita T, Moriyama Y (2011). Effects of a MATE protein inhibitor, pyrimethamine, on the renal elimination of metformin at oral microdose and at therapeutic dose in healthy subjects. Clin PharmacolTher.

[86] Cohen CJ, Andrade-Villanueva J, Clotet B, Fourie J, Johnson MA, Ruxrungtham K (2011). Rilpivirine versus efavirenz with two background nucleoside or nucleotide reverse transcriptase inhibitors in treatment-naive adults infected with HIV-1 (THRIVE): a phase 3, randomised, non-inferiority trial. Lancet.

[87] Molina JM, Cahn P, Grinsztejn B, Lazzarin A, Mills A, Saag M (2011). Rilpivirine versus efavirenz with tenofovir and emtricitabine in treatment-naive adults infected with HIV-1 (ECHO): a phase 3 randomised double-blind active-controlled trial. Lancet.

[88] Kakuda TN, Leopold L, Nijs S, Vandevoorde A, Crauwels HM, Bertelsen KM (2014). Pharmacokinetic interaction between etravirine or rilpivirine and telaprevir in healthy volunteers: a randomized, two-way crossover trial. J Clin Pharmacol.

[89] Zack J, Berg J, Juan A, Pannacciulli N, Allard M, Gottwald M (2015). Pharmacokinetic drug-drug interaction study of ranolazine and metformin in subjects with type 2 diabetes mellitus. Clin Pharmacol Drug Dev.

[90] Deray G, Bochet M, Katlama C, Bricaire F. [Nephrotoxicity of ritonavir]. Presse Med 1998;27(35):1801-3. [French]. 9850700

[91] Burry HC, Dieppe PA (1976). Apparent reduction of endogenous creatinine clearance by salicylate treatment. Br Med J.

[92] Matsui K, Kamijo-Ikemori A, Sugaya T, Ikeda H, Okuse C, Shibagaki Y (2015). Does elevation of serum creatinine in patients with chronic hepatitis C under therapy of telaprevir mean renal impairment?. Nephrology (Carlton).

[93] Suzuki F, Suzuki Y, Sezaki H, Akuta N, Seko Y, Kawamura Y (2013). Exploratory study on telaprevir given every 8 h at 500 mg or 750 mg with peginterferon-alpha-2b and ribavirin in hepatitis C patients. Hepatol Res.

[94] Nakada T, Kito T, Inoue K, Masuda S, Inui K, Matsubara K (2014). Evaluation of the potency of telaprevir and its metabolites as inhibitors of renal organic cation transporters, a potential mechanism for the elevation of serum creatinine. Drug MetabPharmacokinet.

[95] Naderer O, Nafziger AN, Bertino JS Jr (1997). Effects of moderate-dose versus high-dose trimethoprim on serum creatinine and creatinine clearance and adverse reactions. Antimicrob Agents Chemother.

[96] Kastrup J, Petersen P, Bartram R, Hansen JM (1985). The effect of trimethoprim on serum creatinine. Br J Urol.

[97] Myre SA, McCann J, First MR, Cluxton RJ Jr (1987). Effect of trimethoprim on serum creatinine in healthy and chronic renal failure volunteers. Ther Drug Monit.

[98] Nakada T, Kudo T, Kume T, Kusuhara H, Ito K (2018). Quantitative analysis of elevation of serum creatinine via renal transporter inhibition by trimethoprim in healthy subjects using physiologically-based pharmacokinetic model. Drug MetabPharmacokinet.

[99] Pollak PT, Sharma AD, Carruthers SG (1993). Creatinine elevation in patients receiving amiodarone correlates with serum amiodarone concentration. Br J Clin Pharmacol.

[100] Martin P, Oliver S, Kennedy SJ, Partridge E, Hutchison M, Clarke D (2012). Pharmacokinetics of vandetanib: three phase I studies in healthy subjects. Clin Ther.

[101] Zhang Y, Warren MS, Zhang X, Diamond S, Williams B, Punwani N (2015). Impact on creatinine renal clearance by the interplay of multiple renal transporters: a case study with INCB039110. Drug MetabDispos.

[102] Nakada T, Kudo T, Kume T, Kusuhara H, Ito K (2019). Estimation of changes in serum creatinine and creatinine clearance caused by renal transporter inhibition in healthy subjects. Drug MetabPharmacokinet.

[103] Branten AJ, Vervoort G, Wetzels JF (2005). Serum creatinine is a poor marker of GFR in nephrotic syndrome. Nephrol Dial Transplant.

[104] Hirai K, Ihara S, Kinae A, Ikegaya K, Suzuki M, Hirano K (2016). Augmented renal clearance in pediatric patients with febrile neutropenia associated with vancomycin clearance. Ther Drug Monit.

[105] Udy AA, Roberts JA, Shorr AF, Boots RJ, Lipman J (2013). Augmented renal clearance in septic and traumatized patients with normal plasma creatinine concentrations: identifying at-risk patients. Crit Care.

[106] Udy AA, Jarrett P, Lassig-Smith M, Stuart J, Starr T, Dunlop R (2017). Augmented renal clearance in traumatic brain injury: a single-center observational study of atrial natriuretic peptide, cardiac output, and creatinine clearance. J Neurotrauma.

[107] Helal I, Fick-Brosnahan GM, Reed-Gitomer B, Schrier RW (2012). Glomerular hyperfiltration: definitions, mechanisms and clinical implications. Nat Rev Nephrol.

[108] Udy AA, Roberts JA, Boots RJ, Paterson DL, Lipman J (2010). Augmented renal clearance: implications for antibacterial dosing in the critically ill. Clin Pharmacokinet.

[109] Kyle UG, Genton L, Hans D, Karsegard L, Slosman DO, Pichard C (2001). Age-related differences in fat-free mass, skeletal muscle, body cell mass and fat mass between 18 and 94 years. Eur J Clin Nutr.

[110] Hoagland CL, Gilder H, Shank RE (1945). The synthesis, storage, and excretion of creatine, creatinine, and glycocyamine in progressive muscular dystrophy and the effects of certain hormones on these processes. J Exp Med.

[111] Horber FF, Scheidegger J, Frey FJ (1985). Overestimation of renal function in glucocorticosteroid treated patients. Eur J Clin Pharmacol.

[112] Doi K, Yuen PS, Eisner C, Hu X, Leelahavanichkul A, Schnermann J (2009). Reduced production of creatinine limits its use as marker of kidney injury in sepsis. J Am Soc Nephrol.

[113] Whedon GD, Shorr E (1957). Metabolic studies in paralytic acute anterior poliomyelitis I Alterations in nitrogen and creatine metabolism. J Clin Invest.

[114] Bleiler RE, Schedl HP (1962). Creatinine excretion: variability and relationships to diet and body size. J Lab Clin Med.

[115] Crim MC, Calloway DH, Margen S (1975). Creatine metabolism in men: urinary creatine and creatinine excretions with creatine feeding. J Nutr.

[116] Nair S, O’Brien SV, Hayden K, Pandya B, Lisboa PJ, Hardy KJ (2014). Effect of a cooked meat meal on serum creatinine and estimated glomerular filtration rate in diabetes-related kidney disease. Diabetes Care.

[117] Mayersohn M, Conrad KA, Achari R (1983). The influence of a cooked meat meal on creatinine plasma concentration and creatinine clearance. Br J Clin Pharmacol.

[118] Fitch CD, Sinton DW (1964). A study of creatine metabolism in diseases causing muscle wasting. J Clin Invest.

[119] Shannon JA (1935). The renal excretion of creatinine in man. J Clin Invest.

[120] Bauer JH, Brooks CS, Burch RN (1982). Clinical appraisal of creatinine clearance as a measurement of glomerular filtration rate. Am J Kidney Dis.

[121] Dunn SR, Gabuzda GM, Superdock KR, Kolecki RS, Schaedler RW, Simenhoff ML (1997). Induction of creatininase activity in chronic renal failure: timing of creatinine degradation and effect of antibiotics. Am J Kidney Dis.

[122] Davies DF, Shock NW (1950). Age changes in glomerular filtration rate, effective renal plasma flow, and tubular excretory capacity in adult males. J Clin Invest.

[123] Musso CG, Oreopoulos DG (2011). Aging and physiological changes of the kidneys including changes in glomerular filtration rate. Nephron Physiol.

[124] Cockcroft DW, Gault MH (1976). Prediction of creatinine clearance from serum creatinine. Nephron.

[125] Cheung KL, Lafayette RA (2013). Renal physiology of pregnancy. Adv Chronic Kidney Dis.

[126] Sims EA, Krantz KE (1958). Serial studies of renal function during pregnancy and the puerperium in normal women. J Clin Invest.

[127] Moran SM, Myers BD (1985). Course of acute renal failure studied by a model of creatinine kinetics. Kidney Int.

[128] Dabla PK (2010). Renal function in diabetic nephropathy. World J Diabetes.

[129] Christiansen JS, Gammelgaard J, Frandsen M, Parving HH (1981). Increased kidney size, glomerular filtration rate and renal plasma flow in short-term insulin-dependent diabetics. Diabetologia.

[130] Nelson RG, Bennett PH, Beck GJ, Tan M, Knowler WC, Mitch WE (1996). Development and progression of renal disease in Pima Indians with non-insulin-dependent diabetes mellitus Diabetic Renal Disease Study Group. N Engl J Med.

[131] Mogensen CE (1971). Glomerular filtration rate and renal plasma flow in short-term and long-term juvenile diabetes mellitus. Scand J Clin Lab Invest.

[132] McCance RA, Widdowson EM (1939). Functional disorganization of the kidney in disease. J Physiol.

[133] Viberti GC, Bilous RW, Mackintosh D, Keen H (1983). Monitoring glomerular function in diabetic nephropathy A prospective study. Am J Med.

[134] Levey AS, Bosch JP, Lewis JB, Greene T, Rogers N, Roth D (1999). A more accurate method to estimate glomerular filtration rate from serum creatinine: a new prediction equation Modification of Diet in Renal Disease Study Group. Ann Intern Med.

[135] Levey AS, Coresh J, Greene T, Marsh J, Stevens LA, Kusek JW (2007). Expressing the Modification of Diet in Renal Disease Study equation for estimating glomerular filtration rate with standardized serum creatinine values. Clin Chem.

[136] Lempert KD (2019). Probiotics and CKD progression: are creatinine-based estimates of GFR applicable?. Am J Kidney Dis.

[137] Porrini E, Ruggenenti P, Luis-Lima S, Carrara F, Jiménez A, de Vries AP (2019). Estimated GFR: time for a critical appraisal. Nat Rev Nephrol.

[138] Dalton RN (2010). Serum creatinine and glomerular filtration rate: perception and reality. Clin Chem.

[139] Delanaye P, Cavalier E, Pottel H (2017). Serum creatinine: not so simple!. Nephron.

[140] Sandilands EA, Dhaun N, Dear JW, Webb DJ (2013). Measurement of renal function in patients with chronic kidney disease. Br J Clin Pharmacol.

[141] Molitoris BA (2017). Rethinking CKD evaluation: should we be quantifying basal or stimulated GFR to maximize precision and sensitivity?. Am J Kidney Dis.

[142] Teo SH, Endre ZH (2017). Biomarkers in acute kidney injury (AKI). Best Pract Res Clin Anaesthesiol.

[143] Chapron A, Shen DD, Kestenbaum BR, Robinson-Cohen C, Himmelfarb J, Yeung CK (2017). Does secretory clearance follow glomerular filtration rate in chronic kidney diseases? Reconsidering the intact nephron hypothesis. Clin Transl Sci.

[144] Kwon YE, Lee MJ, Park KS, Han SH, Yoo TH, Oh KH (2017). Cystatin C is better than serum creatinine for estimating glomerular filtration rate to detect osteopenia in chronic kidney disease patients. Yonsei Med J.

[145] Opotowsky AR, Carazo M, Singh MN, Dimopoulos K, Cardona-Estrada DA, Elantably A (2019). Creatinine versus cystatin C to estimate glomerular filtration rate in adults with congenital heart disease: results of the Boston Adult Congenital Heart Disease Biobank. Am Heart J.

[146] Filler G, Lee M (2018). Educational review: measurement of GFR in special populations. Pediatr Nephrol.

[147] Brown CS, Kashani KB, Clain JM, Frazee EN (2016). Cystatin C falsely underestimated GFR in a critically ill patient with a new diagnosis of AIDS. Case Rep Nephrol.

[148] Inker LA, Schmid CH, Tighiouart H, Eckfeldt JH, Feldman HI, Greene T (2012). Estimating glomerular filtration rate from serum creatinine and cystatin C. N Engl J Med.

[149] Grubb AO (2000). Cystatin C--properties and use as diagnostic marker. Adv Clin Chem.

[150] Sjöström P, Tidman M, Jones I (2005). Determination of the production rate and non-renal clearance of cystatin C and estimation of the glomerular filtration rate from the serum concentration of cystatin C in humans. Scand J Clin Lab Invest.

[151] Liu BC, Tang TT, Lv LL, Lan HY (2018). Renal tubule injury: a driving force toward chronic kidney disease. Kidney Int.

[152] Ito S, Kusuhara H, Kumagai Y, Moriyama Y, Inoue K, Kondo T (2012). N-methylnicotinamide is an endogenous probe for evaluation of drug-drug interactions involving multidrug and toxin extrusions (MATE1 and MATE2-K). Clin PharmacolTher.

[153] Kato K, Mori H, Kito T, Yokochi M, Ito S, Inoue K (2014). Investigation of endogenous compounds for assessing the drug interactions in the urinary excretion involving multidrug and toxin extrusion proteins. Pharm Res.

[154] Müller F, Pontones CA, Renner B, Mieth M, Hoier E, Auge D (2015). N(1)-methylnicotinamide as an endogenous probe for drug interactions by renal cation transporters: studies on the metformin-trimethoprim interaction. Eur J Clin Pharmacol.

[155] Tsuruya Y, Kato K, Sano Y, Imamura Y, Maeda K, Kumagai Y (2016). Investigation of endogenous compounds applicable to drug–drug interaction studies involving the renal organic anion transporters, OAT1 and OAT3, in humans. Drug MetabDispos.

[156] Chu X, Chan GH, Evers R (2017). Identification of endogenous biomarkers to predict the propensity of drug candidates to cause hepatic or renal transporter-mediated drug-drug interactions. J Pharm Sci.

[157] Imamura Y, Tsuruya Y, Damme K, Heer D, Kumagai Y, Maeda K (2014). 6β-hydroxycortisol is an endogenous probe for evaluation of drug-drug interactions involving a multispecific renal organic anion transporter, OAT3/SLC22A8, in healthy subjects. Drug MetabDispos.

[158] George JA, Gounden V (2019). Novel glomerular filtration markers. Adv Clin Chem.

