# Supplementary material for: Challenges of Serum Creatinine Level in GFR Assessment and Drug Dosing Decisions in Kidney Injury
Source: Adv Pharm Bull. 2024 Dec 8;14(4):745–58. doi: 10.34172/apb.42345 (PMC11970497; doi:10.34172/apb.42345)
Supplement: Supplementary file 1 — Table S1. Enzymes and transporters in creatinine biosynthesis [file apb-14-745-s001.pdf]

***Adv Pharm Bull*.** 2024;14(4):745-S1

doi: 10.34172/apb.42345

https://apb.tbzmed.ac.ir

Supplementary file 1

Table S1. Enzymes and transporters in creatinine biosynthesis

Protein (Abbreviated name)	L-arginine-glycine amidinotransferase (AGAT)	Guanidoacetate methyltransferase (GAMT)	Creatine transporter/ *SLC6A8*	Creatine kinase (CK)
**Tissue Distribution**	Kidneys, Pancreas, Liver (cow, pig, monkey, and human), heart, lung, spleen, muscle, brain, testis, and thymus	Liver, Pancreas, Testis, Caput epididymis, Ovary, spleen, heart, skeletal muscle, lung, kidney	Skeletal muscle, Kidney, Heart, brain	Skeletal muscle, Myocardium, Smooth muscle, Brain, Gastrointestinal tract, Lung, Kidney.
**Substrate**	L-arginine, glycine	Guanidoacetate	Creatine (but not creatinine)	Creatine
**Inhibitor**	L-Ornithine, Creatine, Cyclocreatine, N-acetimidoylsarcosine, N-ethylguanidinoacetate, Estrogens Diethylstilbestrol, Methyltestosterone	/	Creatine, Guanidinoacetate, Guanidinopropionate, 3-guanidinobutyrate, Estrogen, Cycloheximide, Cyclocreatine, Ouabain, Digoxin	Nitric oxide, AMP-activated protein kinase
**Inducer**	Testosterone, Thyroxine, Growth hormone, Testosterone, Estrogen	/	Isoproterenol, Norepinephrine, N6,2′-O-dibutyryladenosine 3′,5′-cyclic monophosphate, Insulin, Insulin-like growth factor I, 3,3',5'-triiodothyronine, Amylin, Testosterone	β-guanidino propionic acid,
**Diet or disease**	Fasting ↓, protein-free diets ↓, Vitamin E deficiency ↓, Streptozotocin-induced diabetes↓, Thyroidectomy ↓, Hypophysectomy ↓, Chronic renal	Hepatocarcinoma↓, Alloxan induced-diabetic↓	Diet containing creatine ↓, 3,3’,5-triiodothyronine-induced creatinuria ↓, Cell energy depletion ↓	Muscle damage and necrosis ↑, Facioscapulohumeral dystrophy↑, Muscular dystrophy↑, Hypothyroidism↑, Infective

	failure ↓			rhabdomyolysis↑, Oxidative and radical damage ↑, Alzheimer's disease↓, Pick's disease↓
**References**	(Hoberman et al., 1948; Van Pilsum and Wahman, 1960; Fitch et al., 1961; Walker, 1979; Funahashi et al., 1981; Van Pilsum et al., 1982; Levillain et al., 1995; Wyss and Kaddurah-Daouk, 2000)	(Van Pilsum et al., 1972; Walker, 1979; Yanokura and Tsukada, 1982; Henderson et al., 1983; Wyss and Kaddurah-Daouk, 2000; Joncquel-Chevalier Curt et al., 2015)	(Gonda and Quastel, 1962; Kurahashi and Kuroshima, 1977; Loike et al., 1988; Bennett et al., 1994; Odoom et al., 1996; Guerrero-Ontiveros and Wallimann, 1998; Steenge et al., 1998; Wyss and Kaddurah-Daouk, 2000; Joncquel-Chevalier Curt et al., 2015)	(Aksenov et al., 1997; Ponticos et al., 1998; Kaasik et al., 1999; Brancaccio et al., 2007)

Aksenov MY, Aksenova MV, Payne RM, Smith CD, Markesbery WR, Carney JM (1997) The expression of creatine kinase isoenzymes in neocortex of patients with neurodegenerative disorders: Alzheimer's and Pick's disease. Exp Neurol 146: 458-465. https://doi.org/10.1006/exnr.1997.6550

Bennett SE, Bevington A, Walls J (1994) Regulation of intracellular creatine in erythrocytes and myoblasts: influence of uraemia and inhibition of Na,K-ATPase. Cell Biochem Funct 12: 99-106. https://doi.org/10.1002/cbf.290120204

Brancaccio P, Maffulli N, Limongelli FM (2007) Creatine kinase monitoring in sport medicine. Br Med Bull 81-82: 209-230. https://doi.org/10.1093/bmb/ldm014

Fitch CD, Hsu C, Dinning JS (1961) The mechanism of kidney transamidinase reduction in vitamin E-deficient rabbits. J Biol Chem 236: 490-492.

Funahashi M, Kato H, Shiosaka S, Nakagawa H (1981) Formation of arginine and guanidinoacetic acid in the kidney in vivo. Their relations with the liver and their regulation. J Biochem 89: 1347-1356. https://doi.org/10.1093/oxfordjournals.jbchem.a133326

Gonda O, Quastel JH (1962) Effects of ouabain on cerebral metabolism and transport mechanisms in vitro. Biochem J 84: 394-406. https://doi.org/10.1042/bj0840394

Guerrero-Ontiveros ML, Wallimann T (1998) Creatine supplementation in health and disease. Effects of chronic creatine ingestion in vivo: down-regulation of the expression of creatine transporter isoforms in skeletal muscle. Mol Cell Biochem 184: 427-437. https://doi.org/10.1023/A:1006895414925

Henderson GD, Xue GP, Snoswell AM (1983) Carnitine and creatine content of tissues of normal and alloxan-diabetic sheep and rats. Comp Biochem Physiol B 76: 295-298.

Hoberman HD, Sims EA, Engstrom WW (1948) The effect of methyltestosterone on the rate of synthesis of creatine. J Biol Chem 173: 111-116.

Joncquel-Chevalier Curt M, Voicu PM, Fontaine M, Dessein AF, Porchet N, Mention-Mulliez K, Dobbelaere D, Soto-Ares G, Cheillan D, Vamecq J (2015) Creatine biosynthesis and transport in health and disease. Biochimie 119: 146-165. https://doi.org/10.1016/j.biochi.2015.10.022

Kaasik A, Minajeva A, De Sousa E, Ventura-Clapier R, Veksler V (1999) Nitric oxide inhibits cardiac energy production via inhibition of mitochondrial creatine kinase. FEBS Lett 444: 75-77. https://doi.org/10.1016/s0014-5793(99)00033-2

Kurahashi M, Kuroshima A (1977) Mechanism of triiodothyronine-induced creatinuria in the rat. Am J Physiol 233: E91-96. https://doi.org/10.1152/ajpendo.1977.233.2.E91

Levillain O, Marescau B, de Deyn PP (1995) Guanidino compound metabolism in rats subjected to 20% to 90% nephrectomy. Kidney Int 47: 464-472. https://doi.org/10.1038/ki.1995.59

Loike JD, Zalutsky DL, Kaback E, Miranda AF, Silverstein SC (1988) Extracellular creatine regulates creatine transport in rat and human muscle cells. Proc Natl Acad Sci U S A 85: 807-811. https://doi.org/10.1073/pnas.85.3.807

Odoom JE, Kemp GJ, Radda GK (1996) The regulation of total creatine content in a myoblast cell line. Mol Cell Biochem 158: 179-188. https://doi.org/10.1007/bf00225844

Ponticos M, Lu QL, Morgan JE, Hardie DG, Partridge TA, Carling D (1998) Dual regulation of the AMP-activated protein kinase provides a novel mechanism for the control of creatine kinase in skeletal muscle. EMBO J 17: 1688-1699. https://doi.org/10.1093/emboj/17.6.1688

Steenge GR, Lambourne J, Casey A, Macdonald IA, Greenhaff PL (1998) Stimulatory effect of insulin on creatine accumulation in human skeletal muscle. Am J Physiol 275: E974-979. https://doi.org/10.1152/ajpendo.1998.275.6.E974

Van Pilsum JF, McGuire DM, Towle H (1982) On the mechanism of the alterations of rat kidney transamidinase activities by diet and hormones. Adv Exp Med Biol 153: 291-297. https://doi.org/10.1007/978-1-4757-6903-6_35

Van Pilsum JF, Stephens GC, Taylor D (1972) Distribution of creatine, guanidinoacetate and the enzymes for their biosynthesis in the animal kingdom. Implications for phylogeny. Biochem J 126: 325-345. https://doi.org/10.1042/bj1260325

Van Pilsum JF, Wahman RE (1960) Creatine and creatinine in the carcass and urine of normal and vitamin E-deficient rabbits. J Biol Chem 235: 2092-2094.

Walker JB (1979) Creatine: biosynthesis, regulation, and function. Adv Enzymol Relat Areas Mol Biol 50: 177-242. https://doi.org/10.1002/9780470122952.ch4

Wyss M, Kaddurah-Daouk R (2000) Creatine and creatinine metabolism. Physiol Rev 80: 1107-1213. https://doi.org/10.1152/physrev.2000.80.3.1107

Yanokura M, Tsukada K (1982) Decreased activities of glycine and guanidinoacetate methyltransferases and increased levels of creatine in tumor cells. Biochem Biophys Res Commun 104: 1464-1469. https://doi.org/10.1016/0006-291x(82)91415-2
